# Towards an Implantable Aptamer Biosensor for Monitoring in Inflammatory Bowel Disease

**DOI:** 10.3390/bios15080546

**Published:** 2025-08-19

**Authors:** Yanan Huang, Wenlu Duan, Fei Deng, Wenxian Tang, Sophie C. Payne, Tianruo Guo, Ewa M. Goldys, Nigel H. Lovell, Mohit N. Shivdasani

**Affiliations:** 1 Graduate School of Biomedical Engineering, UNSW Sydney, Sydney 2052, Australia; yanan.huang@uts.edu.au (Y.H.); w.duan@unsw.edu.au (W.D.); fei.deng@unsw.edu.au (F.D.); wenxian.tang@unibas.ch (W.T.); t.guo@unsw.edu.au (T.G.); e.goldys@unsw.edu.au (E.M.G.); n.lovell@unsw.edu.au (N.H.L.); 2 Tyree Institute of Health Engineering (IHealthE), UNSW Sydney, Sydney 2052, Australia; 3 Department of Chemistry, University of Basel, 4058 Basel, Switzerland; 4 Bionics Institute, Melbourne 3065, Australia; spayne@bionicsinstitute.org; 5 Medical Bionics Department, University of Melbourne, Melbourne 3010, Australia

**Keywords:** aptamer, biosensor, inflammatory bowel disease, cytokine, interleukin-6, hydrogel

## Abstract

Inflammatory bowel disease (IBD) is a relapsing–remitting condition resulting in chronic inflammation of the gastrointestinal tract. Present methods are either inadequate or not viable for continuous tracking of disease progression in individuals. In this study, we present the development towards an implantable biosensor for detecting interleukin-6 (IL-6), an important cytokine implicated in IBD. The optimised sensor design includes a gold surface functionalised with a known IL-6-specific aptamer, integrating a recognition sequence and an electrochemical redox probe. The IL-6 aptasensor demonstrated a sensitivity of up to 40% and selectivity up to 10% to the IL-6 target in vitro. Sensors were found to degrade over 7 days when exposed to recombinant IL-6, with the degradation rate rapidly increasing when exposed to intestinal mucosa. A feasibility in vivo experiment with a newly designed implantable gut sensor array confirmed rapid degradation over a 5-h implantation period. We achieved up to a 93% reduction in sensor degradation rates, with a polyvinyl alcohol–methyl acrylate hydrogel coating that aimed to reduce nonspecific interactions in complex analytes compared to uncoated sensors. Degradation was linked to desorption of the monolayer leading to breakage of gold thiol bonds. While there are key challenges to be resolved before a stable implantable IBD sensor is realised, this work highlights the potential of aptamer-based biosensors as effective tools for long-term diagnostic monitoring in IBD.

## 1. Introduction

Inflammatory bowel disease (IBD), comprising Crohn’s disease (CD) and ulcerative colitis (UC), is characterised by chronic inflammation of the gastrointestinal tract with periods of relapse and remission [1]. The symptoms of CD and UC include abdominal pain, diarrhoea, and rectal bleeding and can severely impact quality of life [1,2,3]. In CD, any part of the gastrointestinal tract from the mouth to the anus can be chronically inflamed, whereas in UC, inflammation is typically confined to the colon (large intestine) and the rectum [4,5]. Although the exact causes of IBD remain unclear, research suggests that a combination of environmental factors and a dysregulated immune response plays a critical role in triggering this autoimmune condition [1,6]. To increase treatment success, it is widely acknowledged in the clinic that a “personalised medicine” approach should be adopted, where decisions are made based on accurate detection and continuous monitoring of validated biomarkers of inflammation [7,8].

Diagnosing and monitoring IBD currently involves a combination of clinical assessments, laboratory tests, imaging studies, and endoscopic procedures [9]. However, they are all limited to a single time point or often inconvenient to collect on a short-term routine basis [10]. This underscores the need for new approaches to monitor intestinal inflammation activity, preferably in near-real time, allowing for timely adjustments in treatment and accurate assessment of disease status. While there is no single biomarker to date that has emerged as a strong surrogate for monitoring intestinal inflammation, recent studies have implicated several biomarkers as important for IBD monitoring [11]. In particular, cytokines, which are chemical messengers essential for cell signalling and the regulation of inflammation, have been suggested to play a pivotal role in IBD and may serve as important diagnostic biomarkers, particularly in combination with other non-cytokine markers such as C-reactive protein and Calprotectin [11]. Recent genetic and immunological studies have directly linked cytokines to the pathogenesis of IBD, with an imbalance between pro-inflammatory cytokines (interleukin-1β, interleukin-6, tumour necrosis factor-α, etc.) and anti-inflammatory cytokines (interleukin-10, interleukin-4, transforming growth factor-β, etc.) driving inflammation, recurrence, and exacerbation [12]. Assessing intestinal-specific inflammation through longitudinal monitoring of cytokines may therefore be useful in IBD [13]. Conventional methods for cytokine detection, such as enzyme-linked immunoassays (ELISAs) [14], chemiluminescent immunoassays (CLIAs) [15], radioimmunoassays (RIAs) [14], and Surface Plasmon Resonance (SPR) [15], would not be viable for long-term monitoring. This is largely due to the requirement of complex equipment, specialised expertise, and, importantly, the inability to provide information about the dynamics of cytokine release within the intestine, something which biosensors could be highly capable of achieving [16].

There have been some recent developments in non-invasive monitoring of cytokines through sweat-based biosensors, with non-invasiveness being the primary appeal. However, such devices are currently limited to 24 h of monitoring after which they need replacement [17]. More importantly, measurements in sweat, while correlated to serum or faecal measurements, may not be sensitive enough to detect small changes in inflammatory processes that, if left unmanaged, could result in a cascade of negative effects. Such devices can result in false positives, as other systemic inflammatory episodes not specific to IBD can cause an increase in sweat biomarkers. As such, monitoring of key cytokine biomarkers within the gut itself may prove to be very useful in managing the disease due to their release through intestinal epithelial cells. Aptamer sensors, often referred to as aptasensors, have desirable characteristics in this regard, as they have the potential capability to perform longitudinal monitoring in vivo without needing replacement due to the reversibility of aptamers [18]. Several aptasensors for in vivo monitoring in various health scenarios, including cytokine monitoring, are currently in development for other health conditions, but none have so far been commercialised [19,20,21,22]. Key to the success of an in vivo aptasensor are its sensitivity, specificity, and stability in target biomarker measurement. In this study, we explored the feasibility of an aptasensor for interleukin-6 (IL-6), one of the important cytokines implicated in IBD [17]. There are studies that have shown overexpression of IL-6 in inflamed bowel tissues of IBD patients [23,24], and more recently, therapies specifically targeting IL-6 inhibition are in clinical trials [25,26]. We tested our sensor’s ability to measure IL-6 and quantified its selectivity and stability in simple and complex in vitro environments. In addition, to improve sensor stability, we tested the effectiveness of a layer of hydrogel covering the exposed surface in prolonging sensor life when exposed to protein-containing analytes in vitro. Finally, as a feasibility pilot study, we designed a sensor electrode array capable of insertion within the large colon and tested this array in an animal model of IBD.

## 2. Materials and Methods

### 2.1. Chemicals

The IL-6 aptamer chosen for this study was commercially synthesised (Integrated DNA Technologies Australia Pty Ltd., Melbourne, Australia) using a known sequence that has been previously reported in the literature to be selective to IL-6 [27,28,29]. The full sequence used is 5′-GG TGG CAG GAG GAC TAT TTA TTT GCT TTT CT-3′ with the 3′-end functionalised with the thiol group and the 5′-end functionalised with methylene blue, a common redox reporter used in aptasensors [30]. Functionalisation with thiol was performed to exploit easy immobilisation of the aptamer onto a gold electrode surface via S-Au bonding. The IL-6 aptamer obtained from the supplier was dissolved in nuclease-free water to a final concentration of 100 µM, aliquoted, and stored at 20 °C prior to use. Other reagents included 6-mercapto-1-hexanol (MCH) used as a surface blocker, tris (2-carboxyethyl) phosphine (TCEP), 98% sulphuric acid (H_2_SO_4_), potassium hexacyanidoferrate (II) (K_4_[Fe(CN)_6_]), and potassium hexacyanoferrate(III) (K_3_[Fe(CN)_6_]), all purchased from Sigma Aldrich (Melbourne, VIC, Australia). Ethanol (EtOH) was purchased from Merck (Darmstadt, Germany). As a supporting electrolyte for all electrochemical measurements, 1× Dulbecco’s phosphate-buffered saline (DPBS) with 0.1 mol L^−1^ NaCl (pH 7.4) was used, for washing electrodes as well as for the preparation and dilution of recombinant protein (Table 1) solutions that were used to challenge sensors. Nuclease-free water was purchased from New England Biolabs Pty Ltd. (Melbourne, VIC, Australia). Au electrodes (3 mm in diameter) were purchased from Ionode Pty Ltd. (Brisbane, QLD, Australia) for benchtop studies; silver–silver chloride (Ag/AgCl) electrodes (as reference electrodes) and platinum wires (as counter electrodes) for electrochemical measurements were purchased from Innovative Instruments Inc. (Tampa, FL, USA); and water purification was performed with a Millipore water system at 18.25 MΩ cm. For the in vivo pilot test, a custom array of 12 gold-ring electrode contacts capable of gentle insertion into the large colon via the rectum was designed and contract manufactured.

### 2.2. Benchtop Sensor Fabrication

**Figure 1:**
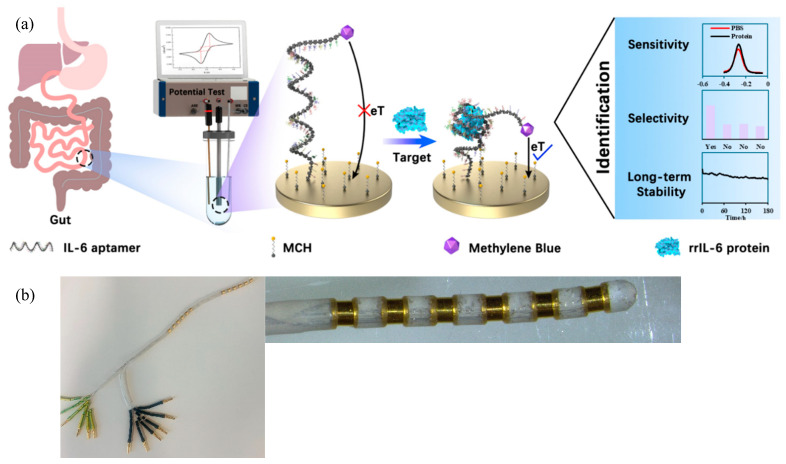
IL-6 aptasensor fabrication and testing. (**a**) Schematic illustration of the fabrication, principle of operation, and testing of our electrochemical aptamer-based IL-6 biosensor. This study focused on assessing sensitivity, selectivity, and long-term stability of the sensor when exposed to simple and complex analytes as well as a preliminary in vivo test for which we (**b**) designed and fabricated an in vivo gut sensor containing 12 ring electrodes.

Sensors were fabricated as previously described in the literature (Figure 1a) [31]. Commercial Au electrodes (3 mm in diameter) were first sonicated in EtOH and Milli-Q water for 5 min each and then electrochemically cleaned in 0.5 M H_2_SO_4_ by application of an oxidising potential of 1.6 V for 5 s followed by a reducing potential of −0.2 V for 10 s and cycling between −0.2 V and 1.6 V for 30 cycles at a scan rate of 0.1 V s^−1^. All electrochemical measurements and electrode cleaning steps were performed using a CHI1040C workstation (CH Instruments Inc., Austin, TX, USA) in a standard three-electrode cell consisting of the sensor as a working electrode, a platinum wire counter electrode, and a Ag/AgCl (3 M KCl) reference electrode. Following electrode cleaning, 1 µL each of the 100 µM IL-6 aptamer stock solution and 50 nM TCEP solution were mixed and kept for 1 h at room temperature, followed by dilution with DPBS to obtain a final desired aptamer concentration. We then immersed freshly cleaned electrodes in this solution overnight at room temperature. Sensors were finally washed with DPBS and then incubated in 5 mM MCH solution (in DPBS) for 4 h at room temperature, before rinsing again with DPBS prior to use. All sensor fabrication steps were carried out in a laminar flow cabinet to minimise contamination. Post fabrication, cyclic voltammetry (CV, −0.1 V to 0.6 V versus Ag/AgCl; 0.1 V/s) tests were performed in 0.5 mM [Fe(CN)_6_]^3/4−^ to confirm whether aptamers were attached to the electrode surface.

### 2.3. In Vivo Sensor Array Design and Fabrication

A hollow tube (99.95% gold, 200 mm length, 2 mm outer diameter, 1.7 mm inner diameter, Goodfellow Cambridge Limited, Huntingdon, UK) was cut into 12 rings (2 mm length) and edges polished. Individual rings were laser welded to the 35N LT DFT wire (Fort Wayne Metals, Fort Wayne, IN, USA), arranged into two groups of 6 electrodes each, then moulded in medical grade silicone (MED 4860 NuSil, Avantor Inc, Radnor, PA, USA), encased in silicone tubing, and oven cured at 120 °C. Each wire ended in a gold pin that allowed electrical connection to individual electrodes (Figure 1b). The total array length was ~9 cm from the tip to the silicone tubing end where all wires exited. This exit point was also where the array exited the rectum during implantation. The array was designed in-house and then manufactured through contract services provided by NeoBionica Pty Ltd. (Melbourne, Australia). Upon receipt of the prototype array, it was subjected to the same cleaning and sensor fabrication steps as described above for the commercial electrodes.

### 2.4. Hydrogel Preparation and Coating

The protective hydrogel coating for this study is based on methacrylated poly (vinyl alcohol) (PVA–MA). Poly (vinyl alcohol) (PVA) is a versatile polymer with tuneable mechanical properties when modified with functional groups. Methacrylation of PVA was achieved by reacting PVA dissolved in dimethyl sulphoxide with 2-isocyanatoethyl methacrylate following a previously published method [32]. The synthesised PVA–MA was then dissolved in DPBS to a final concentration of 10 wt%. Once dissolved, a 0.05 wt% of photo initiator (Irgacure 2959 from a 1% solution) was added to the hydrogel solution. The hydrogel solution was heated to completely dissolve and then cooled to room temperature prior to coating application. For coating, the fabricated aptamer sensor was mounted on a fixture, and a 25 µL drop of the hydrogel solution was applied to the sensor surface. The hydrogel was then polymerised via exposure to ultraviolet light for 3 min at an intensity of 30 mW/cm^2^. After polymerisation, the hydrogel-coated sensor was temporarily stored in DPBS before conducting electrochemical measurements. Hydrogel coating was only performed on benchtop electrodes.

### 2.5. Sensor Interrogation

Electrochemical measurements to interrogate sensors were performed at room temperature using the CHI1040C workstation. Square wave voltammetry (SWV) was performed using a potential window of −0.05 to −0.4 V, a potential step of 0.004 V, and an amplitude of 0.02 V at different frequencies. We interrogated IL-6 aptasensors in various solutions to assess sensitivity, selectivity, and stability. Depending on the test, measurements were either made at a single SWV frequency or by sweeping a range of frequencies from 6 Hz to 500 Hz. For all measurements, the raw difference current between the forward and reverse currents was exported from the system and analysed using custom MATLAB (Release 2024b, Mathworks, Natick, MA, USA) scripts. Background correction was performed on this data and the peak current determined for each measurement. The prototype in vivo sensor array was also tested using CV and SWV in DPBS prior to use in animals. Of the 12 electrodes on the prototype array, ~50% showed measurable signals in DPBS.

### 2.6. Intestinal Mucosa Sample Collection

To assess feasibility towards an in vivo sensor for IBD, we examined the performance of the benchtop sensors in a complex solution that closely resembled the environment of the gut. To prepare this solution, we extracted gut mucosa from a rat subjected to acute colitis as described in our previous work [33]. All animal experiments were approved by the UNSW Animal Care and Ethics Committee (Protocol#21/130A) and complied with the Australian National Health and Medical Research Council code for the care and use of animals for scientific purposes (8th edition, 2013). Acute colitis was induced by injecting 0.25 mL of 1% trinitrobenzenesulphonic acid (TNBS in 50% ethanol, Sigma Aldrich, Melbourne, VIC, Australia) 8 cm into the colon using a custom catheter under isoflurane anaesthesia in a healthy Long Evans rat. Intestinal mucosa was collected hourly over 4–6 h post injection, using filter paper technology validated in humans [34], starting from 30 min post injection of TNBS. For each test, a piece of filter paper (Whatman 42.5 mm, GE Healthcare, Life Sciences, Aurora, OH, USA) wrapped around a custom tube was inserted about 8 cm into the colon, incubated for 20 min, and then removed and transferred to a tube containing 1 mL tris-buffer solution (0.1 M Tris, 0.3% human serum albumin,0.01% sodium azide, and 0.002% tween). Following several measurements, the animal was euthanised with sodium pentobarbital.

### 2.7. In Vitro Sensitivity, Selectivity and Stability Experiments

Separate groups of sensors were fabricated for various benchtop experiments that assessed sensor performance in terms of sensitivity, selectivity to IL-6 versus other non-target proteins, and stability in various analytes. For assessing sensitivity, aptasensors were fabricated using four different aptamer concentrations (n = 3 for each concentration). Sensors were sequentially tested in blank DPBS, followed by a target protein solution (containing 1 ng/mL of IL-6), followed by a second fresh DPBS solution. SWV measurements were conducted at room temperature using a frequency range from 30 to 500 Hz. For each sensor at each frequency, signal gain was calculated as the percentage change in peak current between the protein solution of interest and the peak current measured in blank DPBS. Two separate tests were conducted using different target protein solutions, one prepared with recombinant IL-6 reconstituted in DPBS and another prepared with recombinant IL-6 reconstituted in a calibrator solution (RD-15) obtained from the rat ELISA IL-6 testing kit. The aptamer concentration that yielded the largest signal gain was chosen for subsequent fabrication and testing.

For selectivity assessment, a systematic testing protocol was employed by testing sensors (n = 3) sequentially in various target and non-target protein solutions, alternating with testing in DPBS. All protein concentrations were kept to 0.5 ng/mL (reconstituted in DPBS) except for Human Serum Albumin, which was prepared at a concentration of 10 ng/mL. Each protein solution test was preceded by a DPBS wash (and SWV test) and followed by a DPBS wash (and SWV test) to avoid cross-contamination and confirm that the sensor signal returned to baseline. The procedure was carried out sequentially for a total of 10 different proteins (Table 1), and signal gain was calculated for each protein at each frequency (6–500 Hz) at room temperature. This stepwise approach allowed for the assessment of the sensor’s binding specificity and its ability to distinguish the target protein from other potentially interfering proteins.

For stability assessments, sensors were tested in various analytes for continuous target monitoring, with a measurement made every hour using a frequency of 6 Hz over seven consecutive days. Six groups of sensors (n = 3 each group) were tested in blank DPBS, recombinant rat IL-6 protein solution, and intestinal mucosa solution. For each analyte, one group of sensors was fabricated without a hydrogel coating while the other group was coated with hydrogel. Post testing, analyses of signal peaks were performed as per previous tests and plotted as a function of time. For each sensor, all measurements were normalised to the first time point to better observe changes in sensor signal over time when exposed to each analyte long term. In addition, to compare stability performance across sensor groups, plots of normalised signal strength versus time were fitted by a decaying mathematical function, and the time taken for each sensor to reach a normalised signal value of 0.75 was determined from the fit.

### 2.8. In Vivo Pilot Feasibility Study

For this experiment, two additional healthy Long Evans rats were subjected to the same TNBS injection procedure described above; however, these animals were recovered from anaesthesia and monitored over 48 h, following which a non-recovery experiment was conducted. Once the animal was under a surgical plane of anaesthesia, the sensor array was gently inserted via the rectum ~8–9 cm into the colon, and the gold pins that emanated from the array (Figure 1b) were connected to the CHI1040C potentiostat system. A reference and wire counter electrode were implanted within a subcutaneous pocket in the abdomen. The animal was kept in a supine position throughout the experiment. SWV measurements were made at 5–10 min intervals for a few hours under anaesthesia. Post completion of the experiment, the array was removed and the animal euthanised.

### 2.9. X-Ray Photoelectron Spectroscopy (XPS)

Several studies have shown degradation in aptasensors observed as a loss in measured signal over time, primarily attributed to desorption of the monolayer and loss of oligonucleotides [30,35,36]. Whilst we expected our sensors to also degrade over time, to assess if the mechanism driving degradation was similar, we prepared a new set of samples for an XPS test. We followed the same fabrication protocol as mentioned above but used a 10 mm × 10 mm gold foil (>99.999%, Goodfellow Cambridge Ltd., Huntington, UK) instead of a circular gold benchtop electrode. Three samples were fabricated with one not exposed to any analyte and the other two exposed to either recombinant rat IL-6 protein solution or intestinal mucosa solution for a period of ~60 h. XPS testing was then performed on an Axis Ultra (Kratos Analytical, Manchester, UK) XPS spectrometer with an Al Kα source (1486.6 eV). For each sample, two random spots (0.5 mm diameter) across the surface were scanned and analysed.

## 3. Results and Discussion

### 3.1. Validation of Sensor Quality

Fabricated sensors were examined using CV in 0.5 mM [Fe(CN)_6_]^3/4−^ solution (Figure S1). As expected, freshly cleaned Au electrodes showed quasi-reversible electron transfer behaviour, with the redox couple presenting a minimal peak-to-peak separation (ΔE = 0.1 V) and a maximum current, in both the oxidation and reduction cycles (Figure S1, black line). After IL-6 aptamer immobilisation, electrodes exhibited a decrease in the oxidation and reduction currents as well as larger peak-to-peak separation (ΔE = 0.45 V, Figure S1, red line). This was due to the deposition of MB on the electrode surface, which is less conductive than the original Au substrate, resulting in reduced current flow [37]. Subsequent incubation with MCH (5 mM) was performed to enable passivation of the electrode surface, which has been reported to decrease nonspecific binding to aptasensors [38], presumably by filling in the gold regions left exposed after aptamer assembly (Figure 1a). After MCH incubation, sensors exhibited similar CV curves to aptamer-assembled electrodes (Figure S1, blue line).

### 3.2. Optimisation of Aptamer Concentration and Sensitivity

**Figure 2:**
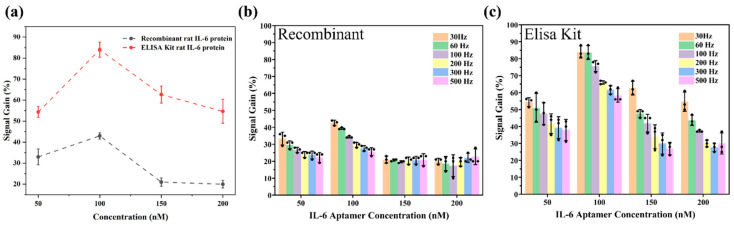
Optimisation of aptamer concentration and sensitivity measurements. (**a**) Signal gain of IL-6 aptasensors (n = 3) for different aptamer concentrations of 50 nM, 100 nM, 150 nM, and 200 nM. Gain was calculated as the % increase in current when exposed to blank DPBS versus a solution containing recombinant rat IL-6 (concentration of 1 ng/mL), which was either reconstituted in DPBS or in the RD-15 calibrator solution obtained from the rat IL-6 ELISA Kit. (**b**,**c**) Signal gain as a function of SWV frequency (30–500 Hz) for different aptamer concentrations when sensors were exposed to a solution containing 1 ng/mL of recombinant rat IL-6 (**b**) reconstituted in DPBS or (**c**) reconstituted in the RD-15 calibrator solution obtained from the rat IL-6 ELISA Kit. Data values shown are mean ± standard deviation with individual data points also shown in (**b**,**c**).

SWV was employed to assess the sensitivity of the biosensors in both blank DPBS and two target solutions (1 ng/mL rrIL-6), where a MB current peak was observed at ~−0.25 V (vs Ag/AgCl). As shown in Figure S2, the SWV signal for MB was significantly elevated in the target protein solution, suggesting successful conformation of the oligonucleotides and a higher concentration of MB molecules positioned closer to the Au surface, resulting in a shorter electron transfer distance (and larger current) compared to a blank DPBS solution. This resulted in a positive value for signal gain. Following this, sensors were fabricated with varying aptamer concentrations (50 nM–200 nM) to determine the optimal response for IL-6 cytokine detection (it should be noted that this 50–200 nM range refers to aptamer concentration during sensor fabrication, not the IL-6 concentration tested, which in all cases was in the pg/mL range). Sensors exhibited the largest signal gain when using an aptamer concentration of 100 nM, both in DPBS-reconstituted (rrIL-6, 1 ng/mL) and ELISA Kit calibrator solution-reconstituted (1 ng/mL) solutions (Figure 2a). Interestingly, sensors exhibited a higher response to the ELISA Kit-prepared solution (mean signal gain > 80% at 30 Hz) compared to the DPBS-based (mean signal gain ~45% at 30 Hz) recombinant protein solution, likely due to additional components in the ELISA Kit RD-15 calibrator, which may have enhanced sensitivity. However, as the full composition of the ELISA Kit is proprietary, a precise explanation for this observation remains speculative at this stage. This result highlights the importance of using pure recombinant proteins reconstituted in DPBS rather than any commercial assays for testing of sensors. Following exposure to proteins, sensors were re-tested in fresh DPBS with the MB peak amplitude returning to baseline (Figure S3) almost immediately, confirming that the IL-6 aptasensor is not only sensitive but also reversible. While IL-6 is a key biomarker in IBD pathogenesis, comprehensive disease monitoring will likely require a multiplexed approach incorporating additional biomarkers such as TNF-α and calprotectin. The present sensor serves as a foundational demonstration of aptamer-based detection within this context.

The selection of frequency in SWV measurements is known to be a critical factor influencing the aptasensor’s sensitivity [39]. Therefore, we further characterised our aptasensors’ performance using different aptamer concentrations at frequencies ranging from 30 to 500 Hz. Consistent with the findings shown in Figure 2a, a 100 nM aptamer concentration resulted in the largest gain when compared at any frequency across the range tested (Figure 2b). In addition, the testing with the ELISA Kit-derived recombinant protein always led to a higher signal gain compared to testing with the PBS-reconstituted recombinant protein. Finally, both DPBS- and ELISA Kit-based solution tests confirmed that lower frequencies led to increased signal gain (Figure 2b,c and Figure S4). Consequently, all subsequent benchtop evaluations of the aptasensors’ properties were conducted using an aptamer concentration of 100 nM.

In terms of IL-6 aptamer concentration optimisation and sensitivity, it is challenging to compare the work in this study to other efforts in aptamer-based sensing of IL-6, as either electrochemically based redox reporters are not used for signal transduction, the aptamer sequence is not the same, aptamers are not used at all, or voltammetry is not used as a measurement technique. Two comparable studies [27,28] that used the same aptamer sequence and similar voltammetry techniques to this work reported a peak signal gain of ~21% over a 0–30 ng/mL [28] and ~77% over a 0–1 ng/mL [27] protein concentration range. The sensitivity of 40% for a 1 ng/mL protein concentration is therefore within the range reported in the literature. However, interestingly, in both previous studies [27,28], signal gain was negative in that they observed a reduction in peak current with increasing protein concentration, in contrast to our study where an increase in peak current was observed. Whilst the sensitivity values recorded in our study are within the range of the two comparable studies, it is important to note that both studies used much higher aptamer concentrations (up to 10 µM) than any of the concentrations used in our work. The other finding that lower frequencies lead to larger signal gain is consistent with some other studies [39], however, the opposite finding also exists in the literature, where higher frequencies lead to larger signal gain in aptasensors [38]. In terms of applicability to IBD, a study by Reinecker et al. [40], measuring concentrations of several biomarkers in isolated cells from colonic biopsies, found that spontaneous IL-6 levels in healthy patients averaged at 57 pg/mL, compared to significantly elevated average levels (5885 pg/mL in UC, 3632 pg/mL in CD) in patients with active IBD and also significantly elevated (2645 pg/mL in UC, 326 pg/mL in CD) in patients with inactive IBD. Based on these findings and our data in Figure 2 and Figure S3, we believe that our sensor should be sensitive enough to able to measure levels in both active and inactive disease, but we cannot ascertain if levels in healthy patients can be detected.

### 3.3. Selectivity of IL-6 Sensors

**Figure 3:**
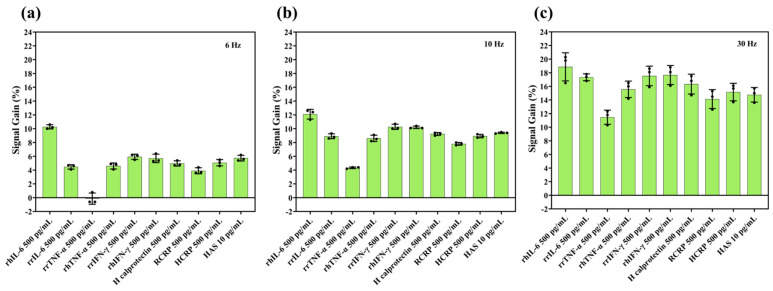
Signal gain of IL-6 aptasensors for each of the target and non-target analytes tested using a frequency of (**a**) 6 Hz; (**b**) 10 Hz; and (**c**) 30 Hz. Data values shown are mean ± standard deviation with individual data points also shown.

For realisation of a sensor that can monitor cytokines such as IL-6 in IBD, it is imperative that the sensor is specific enough in the presence of many other constituents of the gut. As shown in Figure 3, the IL-6 aptasensor was found to be most selective when using a frequency of 6 Hz for measurements. Whilst this is a lower frequency than the one where the maximum sensitivity was measured (30 Hz), this is due to the sensitivity experiments being conducted at an earlier time point and therefore having a different range of frequencies tested. Upon finding that 30 Hz resulted in the highest sensitivity, we decided to extend the frequency range at the lower end for the selectivity experiment. While we did not measure sensitivity at 6 Hz, based on the trend in Figure 2, we would expect it to be either similar or higher than the sensitivity at 30 Hz. The highest selectivity calculated was ~10%, i.e., the difference in signal gain between measurements of recombinant human IL-6 and recombinant rat TNF-α at 6 Hz. For other non-target proteins, selectivity ranged between 2 and 6% for both the low frequencies (Figure 3) and higher frequencies (Figure S5) tested, which is consistent with selectivity values reported for similar electrochemical biosensors in complex biological matrices [28], and reflects the inherent challenges of achieving large discrimination margins in such environments. However, overall, larger signal gains were this time observed at higher frequencies (Figure 3b,c and Figure S5), in contrast to our own sensitivity test above, but it is important to note that all signal gain values in this test were lower than the values in the previous test. This could be related to the protein concentration, as for the initial sensitivity tests, we ran the frequency sweep using a protein concentration of 1 ng/mL whilst for the selectivity tests, we used a 0.5 ng/mL concentration, thus highlighting the interaction between SWV frequency and protein concentration when it comes to signal gain.

The IL-6 aptasensor showed optimal specificity at 6 Hz, indicating that lower frequencies are most effective at minimising sensitivity to non-target cytokines and other biomarkers. Therefore, based on the combined sensitivity and selectivity results, we ran the next set of stability experiments at this frequency. Previous studies that we used to compare our sensitivity results [27,28] reported selectivity values of ~6% and ~56%, once again confirming comparable selectivity to our work, but also highlighting huge variation between studies. In an ideal sensor, selectivity should be close to if not 100%; however, achieving this is extremely unlikely with aptasensors. One possible reason for the lower selectivity may include non-specific adsorption of proteins to the MCH monolayer regardless of the type of protein, which would then interfere with aptamer binding and conformation. Interestingly though, in our work and the work performed by Gao et al. [28], sensitivity and selectivity values were highly comparable and both studies made use of pure gold substrates along with a methylene blue redox reporter to fabricate aptasensors, whilst in the work conducted by Li et al. [27] that demonstrated comparably higher sensitivity and selectivity, they used a nanocomposite carbon nanotube/cobalt hexacyanoferrate/gold nanoparticle surface by modifying a glassy carbon substrate as well as a different electrochemical reporter.

### 3.4. IL-6 Sensor Stability

**Figure 4:**
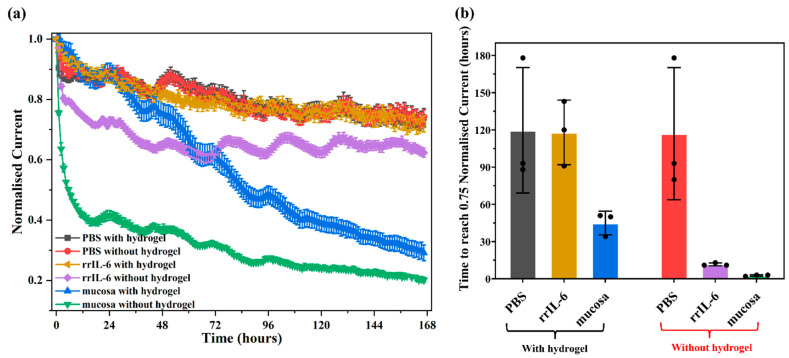
Long-term stability of our IL-6 aptasensors with and without hydrogel coating in various environments using an SWV frequency of 6 Hz. (**a**) IL-6 aptasensors with (n = 3) and without (n = 3) hydrogel coating soaked in blank DPBS, 1 ng/mL rrIL-6 (PBS reconstituted), and intestinal mucosa solution under continuous monitoring for 7 days. Measurements were made every hour; (**b**) time to reach 0.75 normalised current for each batch of IL-6 aptasensors with and without hydrogel coating. Data values shown are mean ± standard deviation with individual data points also shown in (**b**).

**Figure 5:**
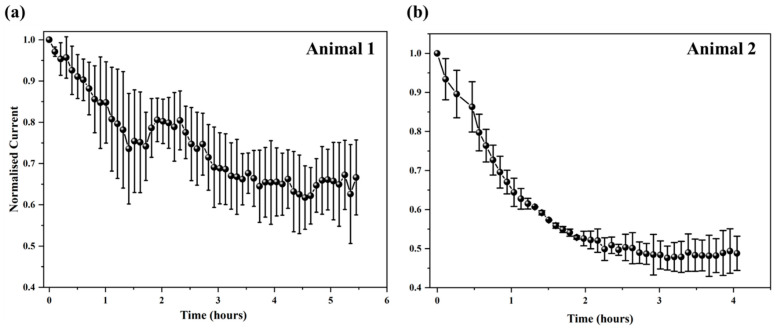
In vivo performance of IL-6 aptasensor array. The sensor array animal 1 (**a**) and animal 2 (**b**) was inserted 8 cm into the colon for 4~5 h, 48 h after inducing inflammation (via TNBS injection). Each data point is the average of three measurements, each on three electrodes. Data values shown are mean ± standard deviation.

Electrochemical-based aptamer biosensors hold significant potential for diagnostic and biomedical applications. However, a critical limitation of this platform is the spontaneous, time-dependent degradation of the bioelectronic interface. This progressive degradation, seen partly as a continuous drop in faradaic current from redox reporters attached to aptamers, limits the in vivo operational life of such sensors to less than 12 h, impeding their long-term use for continuous molecular monitoring in humans [30]. As shown in Figure 4, the IL-6 aptasensor we developed demonstrated high stability in DPBS solution, retaining approximately 72% of the original signal after 7 days of continuous monitoring (Figure 4a, red line, scanned at hourly intervals). However, when aptasensors were immersed in a target IL-6 protein solution for evaluation (Figure 4a, purple line), their signal dropped by 20% within 3 h and continued to decline over the first 3 days. Degradation was further exacerbated when sensors were tested in intestinal mucosa solution, with a 60% signal loss in the first 24 h itself (Figure 4a, green line) followed by further decline over the 7-day period. For the in vivo sensor array, initial SWV tests in DPBS post fabrication revealed only a few electrodes with reliable methylene blue peaks; therefore, only these electrodes were analysed further. In the two animals where the sensor array was tested in vivo, degradation was found to occur most rapidly, with signals dropping by 30–50% in less than 5 h (Figure 5). The decline seen in DBPS, albeit relatively much slower than in solutions with proteins, is most likely due to the desorption of MCH [41] from the electrode surface in highly concentrated deposition solutions [36,42,43]. Additionally, when tested in more complex environments with proteins resembling the intestine, the decline is presumably due to a combination of MCH desorption, aptamer degradation, and non-specific adsorption of cells on the sensor surface, exacerbating the decline [30,35,41,42,43,44,45].

To address the rapid degradation issue, varied methods including kinetic differential measurements [46], the dual-reporter approach [47], and chronoamperometry [48] have been employed and can correct for signal drift and segregate signal changes occurring due to degradation and those occurring due to detection of target biomarkers. While these approaches provide good measurement accuracy and baseline stability for in vivo runs up to several hours, they become ineffective when sensor degradation significantly lowers the signal-to-noise ratio. Moreover, these “corrective” methods do not address the underlying cause of sensor degradation that, as observed by us and others [45], can be clearly exacerbated in complex protein environments. This highlights the need to develop new strategies to enhance the stability of thiol-based self-assembled monolayers by protecting the sensor from “biofouling”. Here, we propose a complementary technology: using a hydrogel matrix to shield sensors from non-specific adsorptions and degradation by restricting access of high-molecular-weight biological components (e.g., large proteins, blood cells) to the sensor surface (Figures S6 and S7).

As shown in Figure 4b, the degradation rate in DPBS in the hydrogel-coated IL-6 aptasensors was similar to the biosensors without hydrogel,; however, when aptasensors were immersed in a 1 ng/mL rrIL-6 protein solution, hydrogel-coated sensors showed a significantly reduced degradation rate, which was ~90% lower than uncoated sensors. Further testing of sensors in the intestinal mucosa solution showed that hydrogel-coated sensors took ~2 days for the signal to degrade by 25% compared to uncoated sensors, which declined rapidly. These findings overall showed a 93.3% enhancement in sensor performance with the hydrogel coating, suggesting that this approach is highly promising towards developing long-term monitoring in IBD [45]. Whilst the use of hydrogels to protect aptamer sensors is not novel [44], to our knowledge, this is the first study to use a PVA–MA hydrogel for an IL-6 aptasensor and demonstrate significantly improved performance in stability. Our results are highly comparable to a recent study [44] that also employed aptasensor protection using a hydrogel (3% agarose) for the detection of kanamycin, in that unprotected sensors took only ~2 h to display a signal loss of 25%, whereas gel-protected sensors lasted for the full 10 h when exposed to whole blood. Other non-hydrogel biofouling strategies for other types of implantable biosensors have also been proposed [49]; however, their applicability to aptasensors specifically is unknown. Based on the data shown in Figure 4b for intestinal mucosa solution, we would expect that our sensor with the PVA–MA hydrogel may last for up to 7–8 days in vivo within the gut, although this is yet to be confirmed. Regarding biocompatibility, it is important to note that the PVA hydrogel we used in this study has been shown to be stable for up to 8 weeks of implantation in other applications [50]. An uncoated sensor, as shown in Figure 5, would have very limited use if any in vivo as it would be subject to significant biofouling. However, even with hydrogel protection, we do acknowledge that other factors such as different pH and temperature conditions in vivo will also ultimately play a significant role in determining sensor lifetime [51].

### 3.5. Determining Sensor Degradation Mechanisms

**Figure 6:**
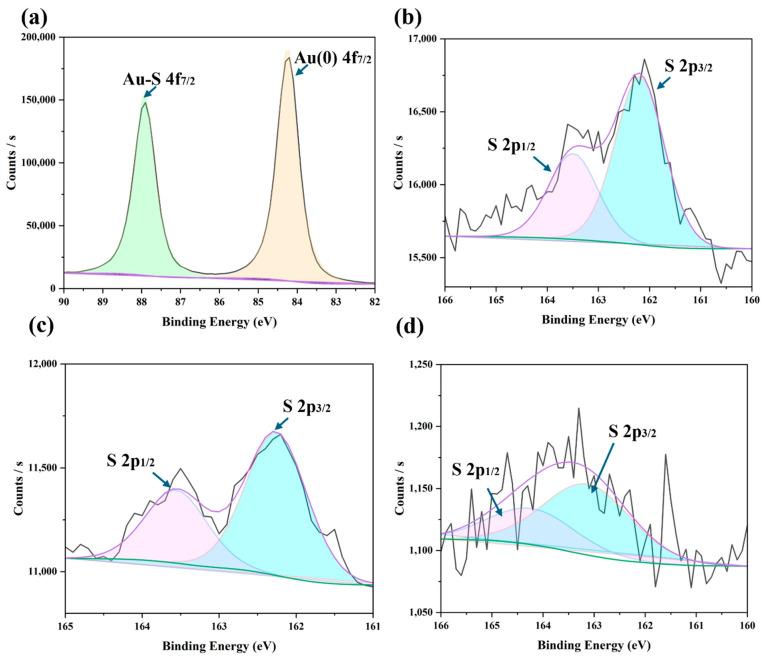
XPS survey patterns of samples including (**a**) Au and (**b**) S for a fresh IL-6 aptasensor; (**c**) S of an IL-6 aptasensor after a 48-h exposure to rrIL-6 protein solution; and (**d**) S of an IL-6 aptasensor after a 48-h exposure to mucosa solution.

To confirm if the degradation mechanism in our sensors during long-term monitoring agreed with the literature, XPS analysis was conducted on new samples that were fabricated under the same conditions as the ones used for electrochemical measurements. Figure S8 shows the XPS survey scan of one sensor surface that was not subjected to any analyte, revealing the presence of Au (24.48%), S (2.19%), C (41.68%), N (1.97%), O (22.76%), Na (1.47%), and Al (5.47%). Detailed spectra for Au and S from this freshly prepared IL-6 aptasensor are shown in Figure 6a,b. XPS peaks corresponding to Au(0) (84.3 eV) and Au-S (87.9 eV) confirmed the formation of the Au–S bond (Figure 6a). Further observation of the S 2p_1/2_ and S 2p_3/2_ peaks (Figure 6b) indicated the binding of sulphanyl sulphur to the Au surface, verifying the aptamer’s successful immobilisation. After two days of passive exposure to rrIL-6 and mucosa solutions, XPS analysis was repeated. The sulphur spectra for these samples (Figure 6c,d) aligned with those of the fresh aptasensor, confirming the persistent Au–S interaction. However, the atomic percentage of sulphur significantly decreased, dropping to 0.96% when exposed to rrIL-6 and 0.08% when exposed to mucosa solutions. These findings suggest that exposure to analytes containing proteins led to detachment of the aptamer, likely due to MCH desorption, with the most significant loss observed in the sample exposed to the complex mucosa environment. Over two days of exposure, the sulphur content in the sample exposed to the mucosa solution decreased by approximately 96.4% compared to that in the freshly prepared sample, correlating with substantial degradation. The accelerated degradation in mucosa solution is attributed to extensive non-specific protein binding to the MCH monolayer, which likely disrupted the aptamer–Au interaction. Supporting evidence is observed in the C and N spectra (Figure S9), where the mucosa-exposed aptasensor showed a pronounced C-N peak at 286.4 eV and a new N-C-N peak at 401.5 eV. These peaks suggest the presence of non-target proteins interfering with the aptamer’s stability, further elucidating the degradation mechanism. In addition to passive degradation, one cannot rule out that our sensors were also subject to active degradation caused by repeated SWV measurements, known to occur in other similar sensors [35]. While we limited our measurement interval to one hour to obtain a sizeable number of measurements in a reasonable time frame, in an IBD patient situation, it would perhaps be sufficient to only scan with the sensor once every 24 h, which would still be significantly more useful in tracking disease patterns compared to currently available techniques. In addition, as suggested by our study, the use of hydrogels (or suitable alternatives) as a protective layer would further prolong sensor life, although the inherently weak Au–S bond may ultimately require further consideration of this paradigm for future aptasensors [52].

Given the vulnerability of unmodified aptamers to enzymatic degradation in mucosal environments, future improvements could include the use of chemically stabilised aptamers (e.g., 2′-F, 2′-OMe modifications) or synthetic analogues (e.g., LNA, PNA) to enhance biosensor durability and functional lifespan in vivo. While our current platform demonstrates promising short-term performance, translation to practical use will require substantial improvements in long-term sensor stability, biofouling resistance, and device integration. Future work will focus on extending operational lifetime to clinically relevant timescales, potentially through advanced hydrogel chemistries, encapsulation materials, and surface passivation strategies.

## 4. Conclusions

We have characterised an aptasensor designed for detection of IL-6, an important cytokine biomarker implicated in IBD prognosis, and designed a sensor array that can be implanted within the lumen of the large intestine in a rat. Sensors fabricated using an IL-6 aptamer with 3′-end thiol and 5′-end methylene blue modifications demonstrated good sensitivity in both recombinant rat and ELISA Kit IL-6 protein solutions, with the highest sensitivities recorded at lower frequencies. Notably, the optimised aptasensor with a 100 nM aptamer concentration when operated at 6 Hz exhibited the highest selectivity to the target cytokine compared to tests at other frequencies. Additionally, we validated our aptasensor’s long-term stability by testing it in PBS, recombinant protein, and mucosa solution and in vivo. Sensor degradation occurred in all cases, with the highest rates of degradation found in vivo. Sensors coated with PVA–MA hydrogel displayed significantly reduced degradation rates, with an approximately 90% reduction in degradation rate when tested in protein solutions and a 93.3% reduction in degradation rate within complex mucosa solutions compared to uncoated sensors. Overall, these results indicate that an IL-6 sensor could be realised for continuous disease monitoring in IBD; however, it is obvious that significant and numerous challenges lay ahead in achieving this outcome. Future work will address additional factors critical to translational application, including cytotoxicity, extended reproducibility testing, the effects of pH and temperature on performance, and storage stability, to enable a more comprehensive assessment of the platform. Such studies will also need to characterise a detection limit and establish a full dose–response curve to ultimately realise a robust sensor architecture tailored for long-term implantation and practical biosensing applications, as our study prioritised signal stability, anti-fouling performance, and IL-6 discrimination under physiologically relevant conditions. The results reported herein also challenge conventional wisdom regarding monolayer aptasensor stability on gold surfaces and shed important light on several previous conclusions that were based on results from shorter-term sensor operation of just a few hours.

## Figures and Tables

**Table 1:** Target and non-target proteins used to test the sensitivity and selectivity of IL-6 aptasensors. [1] In Vitro Technologies (Sydney, NSW, Australia); [2] Abcam Limited (Cambridge, UK); [3] Thermo Fisher Scientific Australia Pty Ltd. (Scoresby, VIC, Australia); [4] Science Limited (Wellington, New Zealand).

Protein Name	Host Species	Abbreviation	Purpose	Supplier
Recombinant IL-6	Human	rhIL-6	Target	[1]
Recombinant IL-6	Rat	rrIL-6	Target	[2]
Recombinant Interferon-gamma (IFN-γ)	Human	rhIFN-γ	Non-target	[1]
Recombinant IFN-γ	Rat	rrIFN- γ	Non-target	[1]
Recombinant Tumour Necrosis Factor-Alpha (TNF-α)	Human	rhTNF-α	Non-target	[1]
Recombinant TNF-α	Rat	rrTNF-α	Non-target	[2]
IL-6 ELISA Kit	Rat	N/A	Target	[3]
Calprotectin	Human	N/A	Non-target	[4]
C-Reactive Protein	Human	hCRP	Non-target	[1]
C-Reactive Protein	Rat	rCRP	Non-target	[2]
Human albumin serum	Human	HAS	Non-target	[1]

## Data Availability

Dataset available on request from the authors.

## Supplementary Materials

The following supporting information can be downloaded at: https://www.mdpi.com/article/10.3390/bios15080546/s1.
