# Supplementary material for: Towards an Implantable Aptamer Biosensor for Monitoring in Inflammatory Bowel Disease
Source: Biosensors (Basel). 2025 Aug 19;15(8):546. doi: 10.3390/bios15080546 (PMC12384986; doi:10.3390/bios15080546)
Supplement: Supplementary file 1 [file biosensors-15-00546-s001.zip › biosensors-3795064-supplementary.pdf]

biosensors

Supplementary Materials

Towards an Implantable Aptamer Biosensor for Monitoring in Inflammatory Bowel Disease

Yanan Huang [1], Wenlu Duan [1], Fei Deng [1,2], Wenxian Tang [1,3], Sophie C. Payne [4,5], Tianruo Guo [1,2], Ewa M. Goldys [1], Nigel H. Lovell [1,2], Mohit N. Shivdasani [1,2,*]

[1] Graduate School of Biomedical Engineering, UNSW Sydney, Sydney 2052, Australia; yanan.huang@uts.edu.au (Y.H.); w.duan@unsw.edu.au (W.D.); fei.deng@unsw.edu.au (F.D.); t.guo@unsw.edu.au (T.G.); e.goldys@unsw.edu.au (E.M.G.); n.lovell@unsw.edu.au (N.H.L.)
[2] Tyree Institute of Health Engineering (IHealthE), UNSW Sydney, NSW 2052, Australia
[3] Department of Chemistry, University of Basel, Basel 4058, Switzerland; wenxian.tang@unibas.ch (W.T.)
[4] Bionics Institute, Victoria 3065, Australia; spayne@bionicsinstitute.org
[5] Medical Bionics Department, University of Melbourne, Victoria 3010, Australia
* Correspondence: m.shivdasani@unsw.edu.au

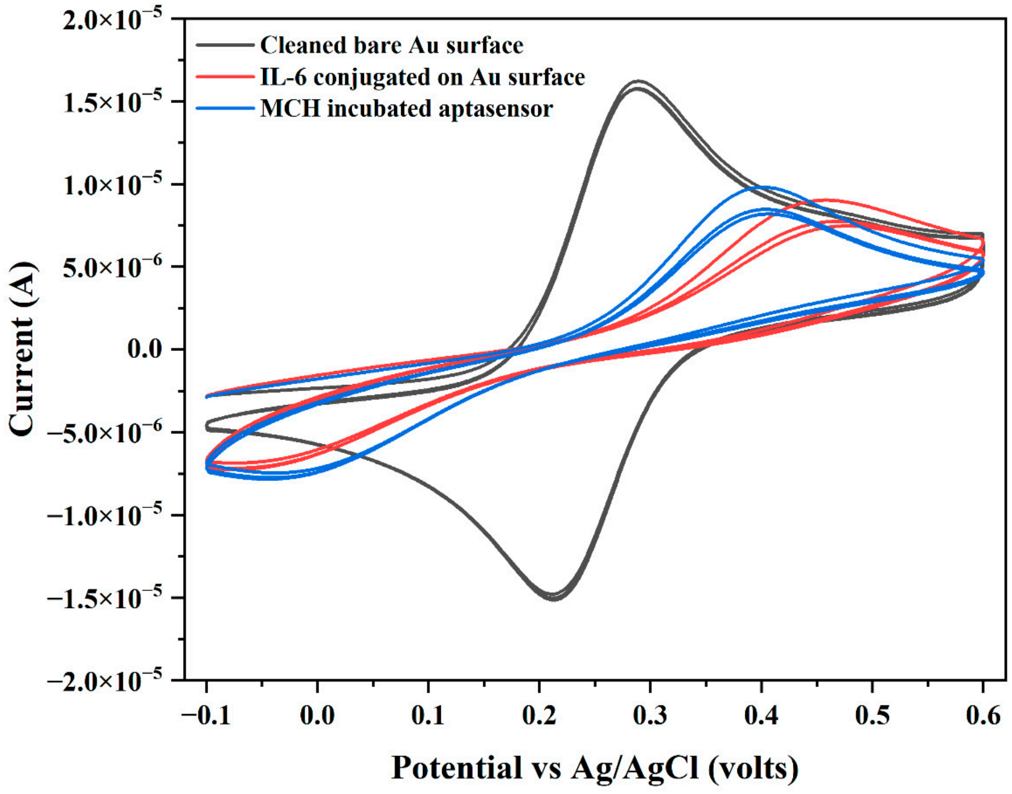

Figure S1. Cyclic voltammetry (CV) test of a cleaned gold electrode, then repeated after IL-6 aptamer immobilisation and after MCH incubation.

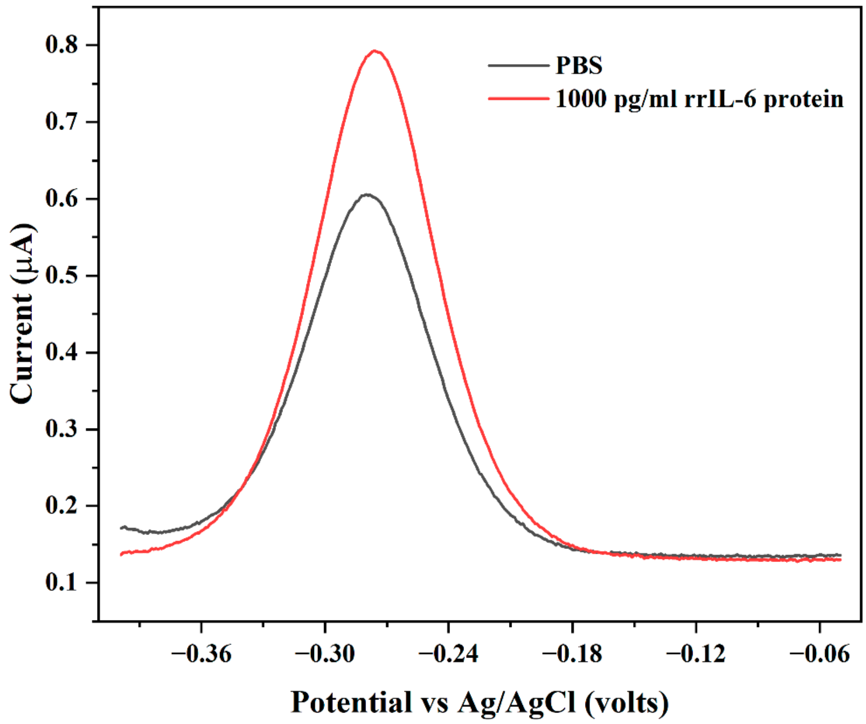

Figure S2. Square wave voltammetry (SWV) test in PBS and a target solution with recombinant rat IL-6 (1000 pg/mL). The peak current was found at ~-0.26 V as expected for a MB redox reporter. A clear increase in current was observed when the sensor was tested in the target solution. SWV frequency was 30 Hz.

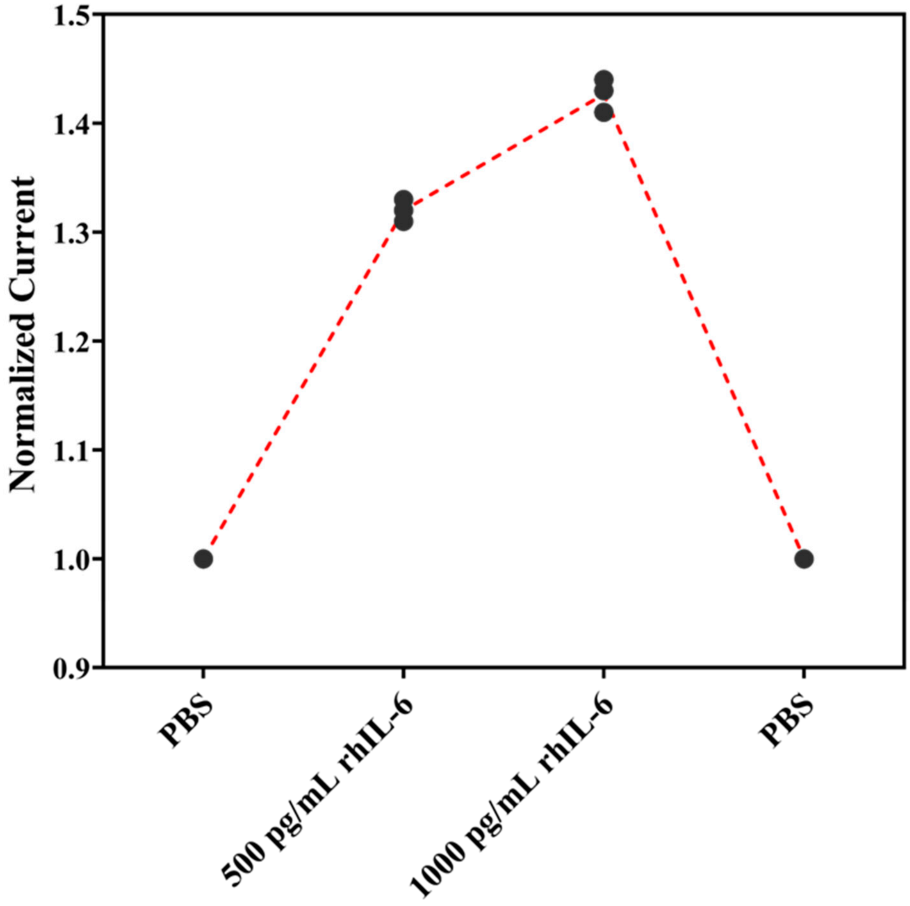

Figure S3. IL-6 aptasensors (n=3) tested for sensitivity and reversibility in PBS, 500 pg/ml and 1000 pg/ml recombinant human IL-6 (rhIL-6) protein solutions. In this experiment, SWV frequency was 30 Hz and the aptamer concentration was 100 nM. Data values shown are mean ± standard deviation.

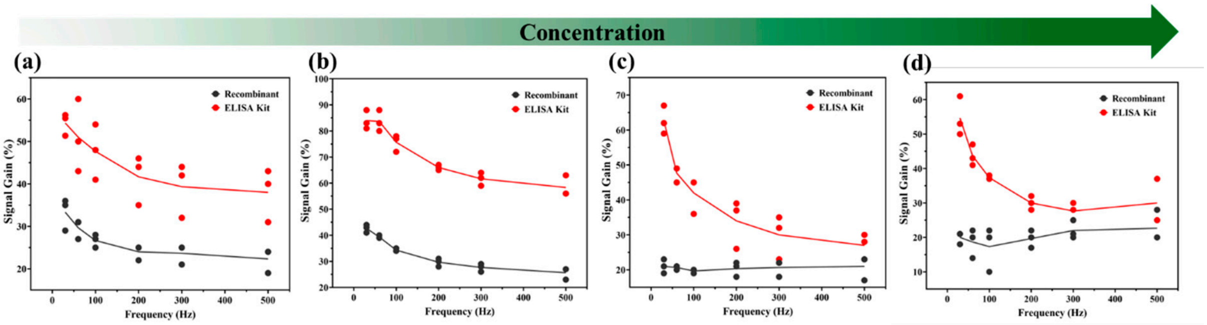

Figure S4. Signal gain for IL-6 aptasensors (n = 3) as a function of SWV test frequency for all aptamer concentrations tested; (a) 50 nM, (b) 100 nM, (c) 150 nM, and (d) 200 nM. In all cases, signal gains were higher when using ELISA kit derived proteins versus recombinant proteins. Data values shown are mean ± standard deviation.

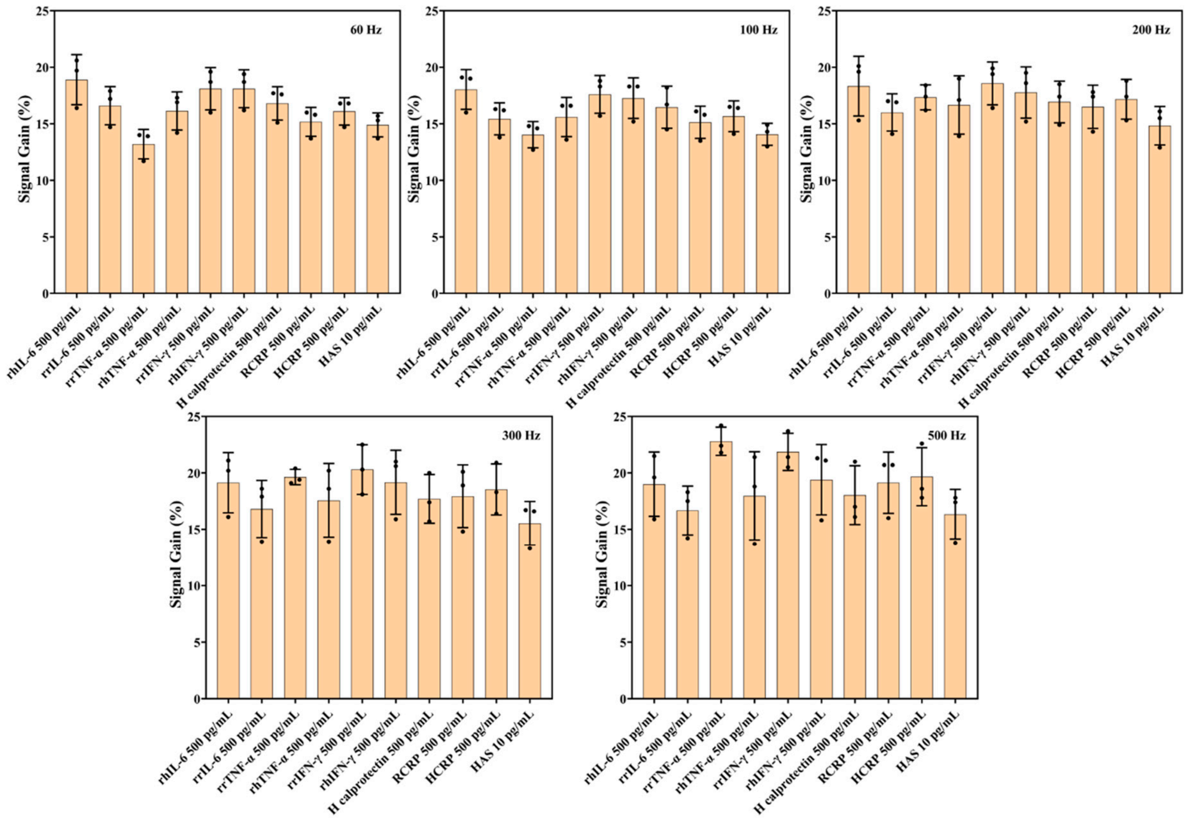

Figure S5. Sensitivity of IL-6 aptasensors (n = 3; 100 nM aptamer concentration) to various target and non-target proteins at different SWV test frequencies from 60-500 Hz. Data values shown are mean ± standard deviation.

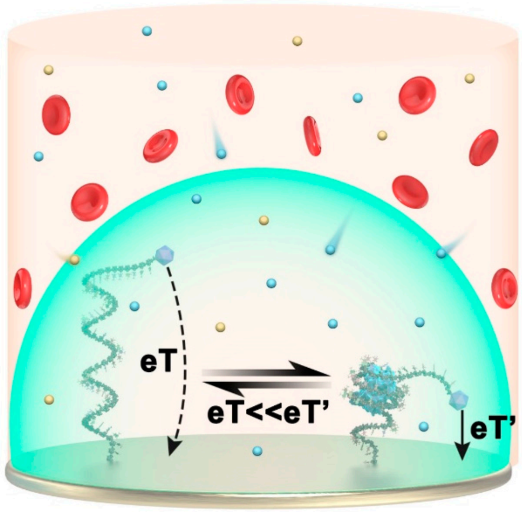

Figure S6. Schematic of proposed hydrogel matrix protecting the IL-6 aptasensor when challenged in complex biological environments.

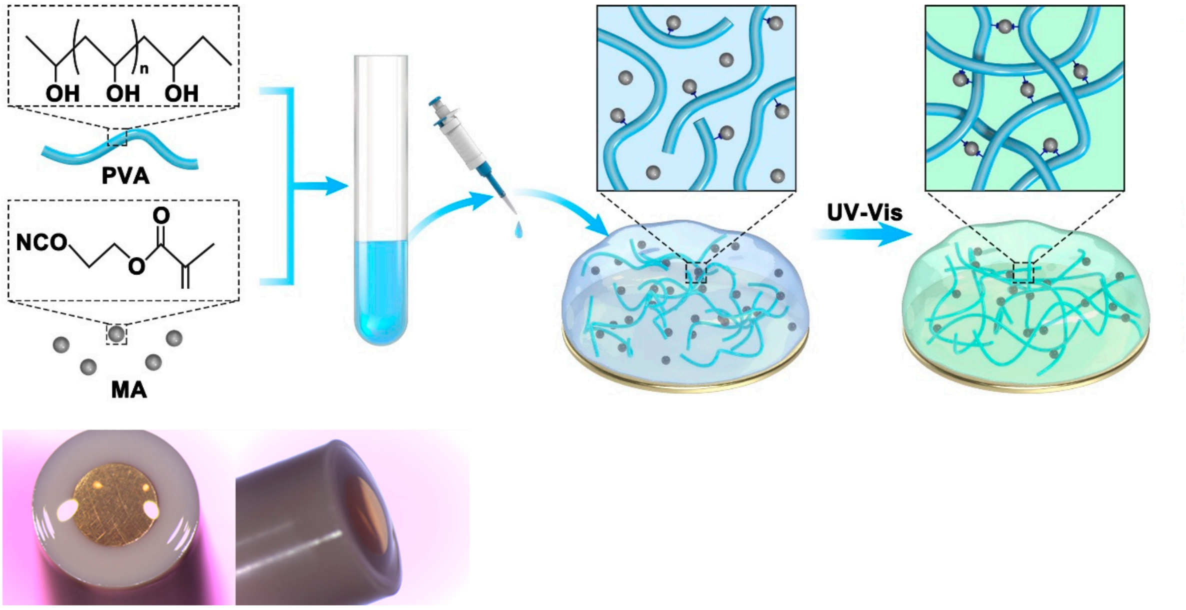

Figure S7. The PVA-MA hydrogel synthesis mechanism. After application of the hydrogel solution to the sensor, exposure to UV light is performed after to enable hydrogel polymerisation. The hydrogel appears as a clear film post-fabrication as seen in the photos.

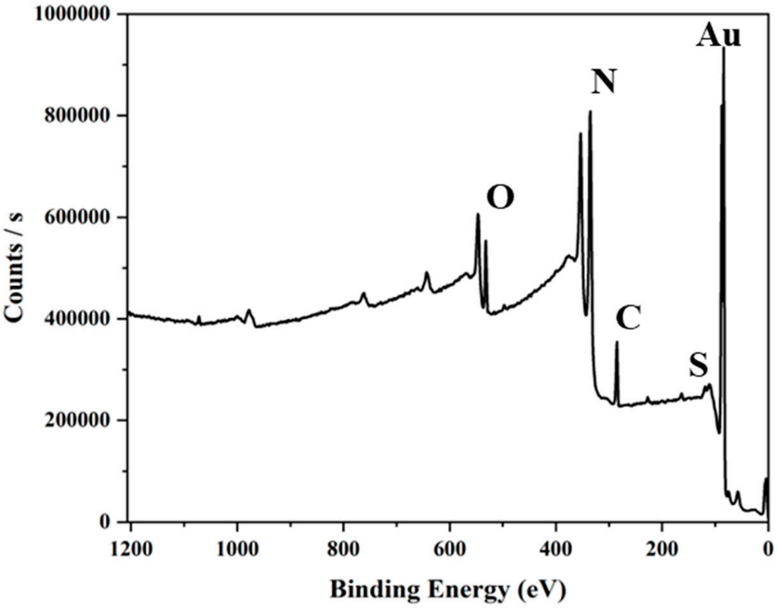

Figure S8. XPS survey spectra of a fresh IL-6 aptasensor.

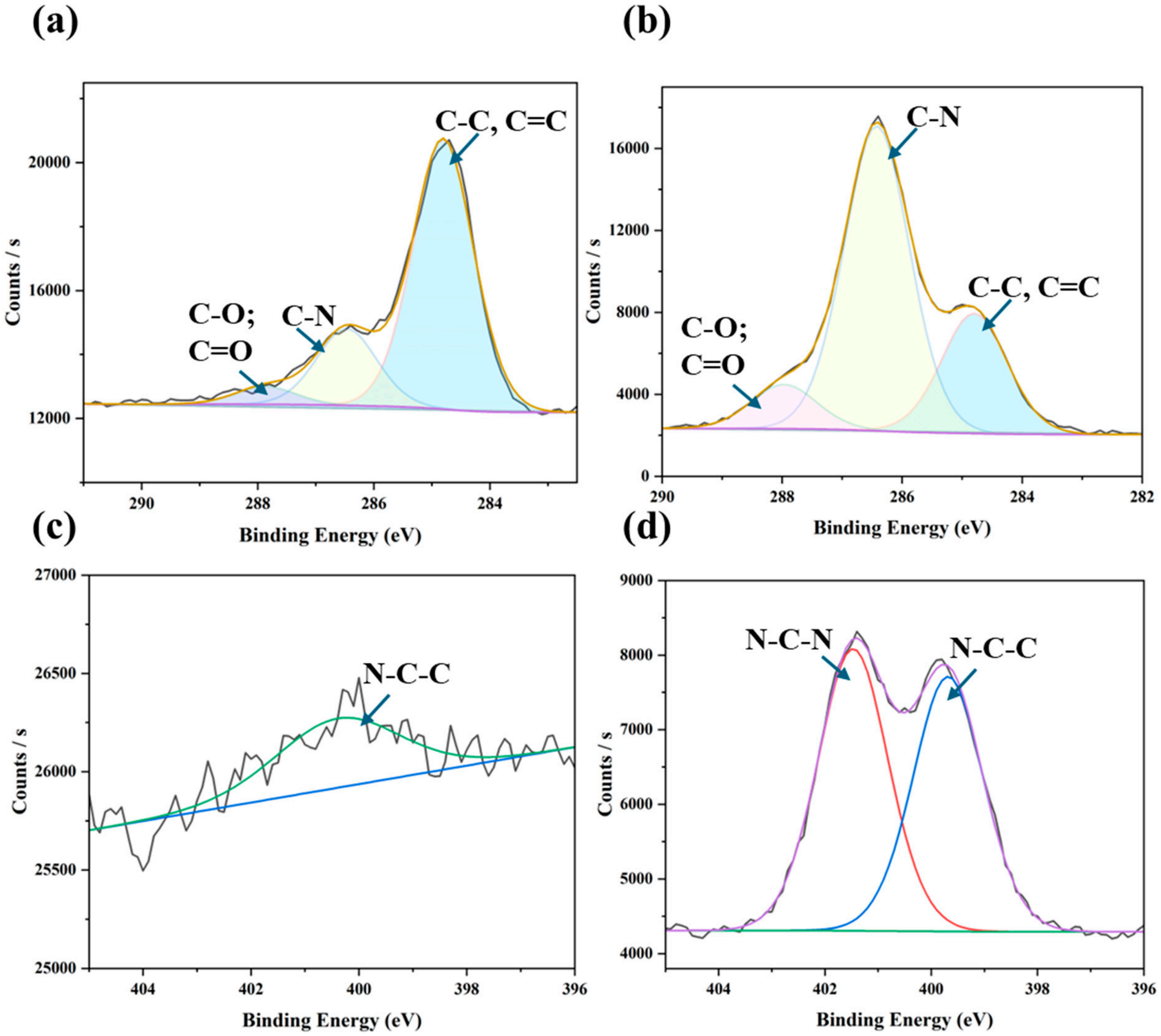

Figure S9. XPS survey patterns of samples including (a) C and (c) N for an IL-6 aptasensor after exposure to IL-6 protein solution; (b) C and (d) N for an IL-6 aptasensor exposed to intestinal mucosa solution.
